# Art of imaging: intersection of aesthetics and analysis in radiology

**DOI:** 10.1093/radadv/umaf032

**Published:** 2025-09-16

**Authors:** Susanna I Lee, Jorge A Soto

**Affiliations:** Massachusetts General Hospital, Harvard Medical School; Boston University School of Medicine

“Creativity is intelligence having fun.”—Albert Einstein

**Figure 1.**
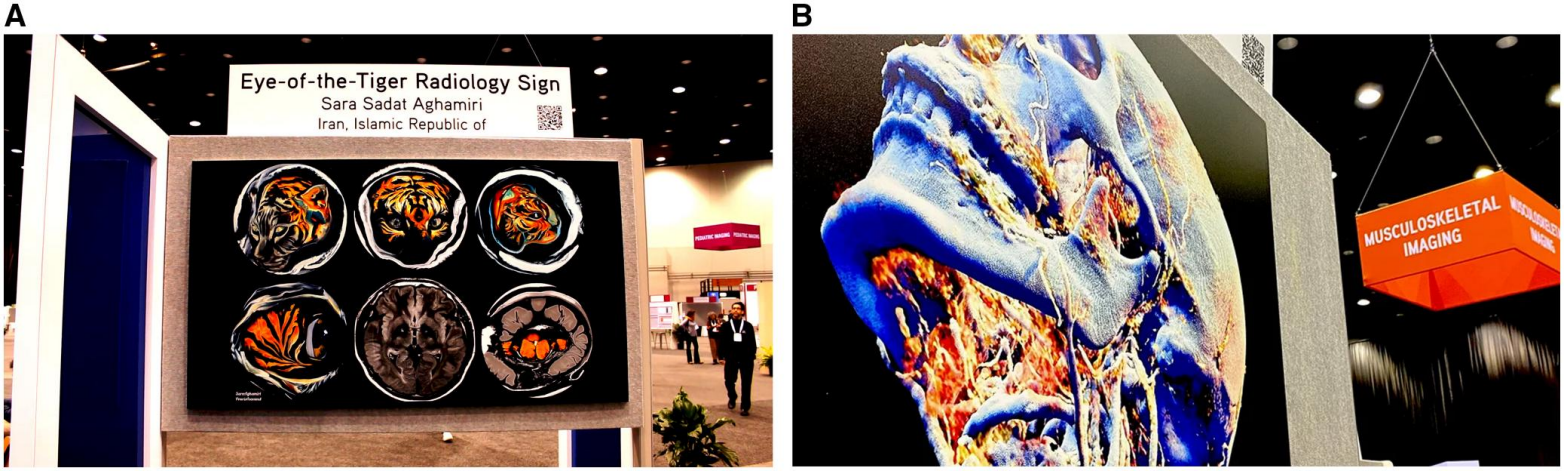
Art of Imaging displays at the RSNA annual meeting (A, B).

Each year, the Radiological Society of North America (RSNA) runs a radiology-themed art competition, Art of Imaging, that showcases the inventive powers of its community and illustrates the multitude of connections between the imaging sciences and the visual arts. Submissions are accepted in a wide range of creative works such as AI-generated pieces, altered medical images, and mixed computer- and manually generated graphics. Winners are selected through public online voting and their work displayed at the annual meeting (Figure 1). This contest has proven immensely popular, garnering hundreds of submissions and thousands of votes each round.

*Radiology Advances* has published a selection of the winning entries as an article over the past year.^1^^,^^2^^,^^3^^,^^4^ Each journal issue contains an Art of Imaging piece that illustrates the original work itself and also includes a brief biography of its creator. In doing so, the journal’s editors wished to celebrate the range of participants that contribute to our profession (eg, technologists, nurses, physicists, trainees) and explore their individual unique interactions with medical imaging. Moreover, the series is intended to highlight the important role that imagination and original thinking play in meaningful radiologic investigation.

Radiology is a particularly technical discipline, with large doses of physics, engineering, and computation at its core. Precision, quantitation, and reproducibility are the standards by which our performance is judged. Radiologic practice involves acquiring high quality images, visual analysis that is meticulous as well as systematic, and standardized reporting. Collection of trustworthy data requires methodical adherence to study procedures and unvaried repetition. Thus, whether working in the clinical or research domain, one can lose sight of the truism that radiologic inquiry is fundamentally an imaginative endeavor. Our goals as investigators are to push the boundaries of radiologic knowledge and practice beyond established paradigms and deploy them clinically to achieve better outcomes for our patients.

The vast majority of this journal’s content is a testament to the scientific rigor that underlies and drives our profession. Yet the boundaries and horizons of our work are defined by what we can envision and choose to build. In this, Art of Imaging is intended as a reminder—an interlude. We ask the reader to step back, explore the visual world aesthetically rather than analytically, and appreciate the ambiguities of our reality.

The 2025 Art of Imaging competition is ongoing. The submissions are in, and the public solicitation for votes closes in mid-September. The editors of *Radiology Advances* look forward to seeing the display of the winning artworks at the society’s annual meeting and to publishing them in the coming year. The themes that emerge are sure to reflect the ideas, concerns, and enthusiasms that we share as a community.
